# Food and Drug Administration approvals in phase 3 Cancer clinical trials

**DOI:** 10.1186/s12885-021-08457-5

**Published:** 2021-06-12

**Authors:** Joseph Abi Jaoude, Ramez Kouzy, Marc Ghabach, Roshal Patel, Dario Pasalic, Elie Ghossain, Austin B. Miller, Timothy A. Lin, Vivek Verma, C. David Fuller, Vivek Subbiah, Bruce D. Minsky, Ethan B. Ludmir, Cullen M. Taniguchi

**Affiliations:** 1 The University of Texas MD Anderson Cancer Center, 1515 Holcombe Blvd Unit 1422, Houston, TX 77030 USA; 2 The University of Texas Health Science Center McGovern Medical School, Houston, TX USA; 3 The Johns Hopkins University School of Medicine, Baltimore, MD USA; 4 The University of Texas MD Anderson Cancer Center, 1515 Holcombe Blvd Unit 1050, Houston, TX 77030 USA

**Keywords:** Oncology, Clinical trials, FDA, Primary endpoint, Surrogate endpoint, Industry

## Abstract

**Background:**

Phase 3 oncologic randomized clinical trials (RCTs) can lead to Food and Drug Administration (FDA) approvals. In this study, we aim to identify trial-related factors associated with trials leading to subsequent FDA drug approvals.

**Methods:**

We performed a database query through the ClinicalTrials.gov registry to search for oncologic phase 3 RCTs on February 2020. We screened all trials for therapeutic, cancer-specific, phase 3, randomized, multi-arm trials. We then identified whether a trial was used for subsequent FDA drug approval through screening of FDA approval announcements.

**Results:**

In total, 790 trials were included in our study, with 225 trials (28.4%) generating data that were subsequently used for FDA approvals. Of the 225 FDA approvals identified, 65 (28.9%) were based on trials assessing overall survival (OS) as a primary endpoint (PEP), two (0.9%) were based on trials with a quality of life (QoL) PEP, and 158 approvals (70.2%) were based on trials with other PEP (*P =* 0.01). FDA approvals were more common among industry funded-trials (219, 97.3%; *P* < 0.001), and less common among trials sponsored by national cooperative groups (21, 9.3%; *P* < 0.001). Finally, increased *pre-hoc* power and meeting patients’ accrual target were associated with FDA approvals (*P* < 0.001).

**Conclusions:**

The majority of FDA approvals are based on data generated from trials analyzing surrogate primary endpoints and trials receiving industry funding. Additional studies are required to understand the complexity of FDA approvals.

## Background

Phase 3 randomized controlled trials (RCTs) are widely regarded as the “gold standard” of evidence to support and shape clinical practice, and are instrumental in Food and Drug Administration (FDA) [1]. As such, the choice of primary endpoints (PEPs) in cancer trials is of paramount importance. Overall survival (OS) and quality of life (QoL) endpoints have been considered to be patient-centered endpoints that are of intrinsic value to patients [2]. Modern trials rely on surrogate endpoints to indirectly predict disease control, OS, and/or QoL [2]. The most common surrogate endpoint used in modern oncology trials is progression-free survival (PFS), which relies on radiographically-identified tumor growth; however, PFS has been found to have limited predictive value for both OS and QoL [3, 4]. The rising use of surrogate endpoints and the increased cost of novel oncology treatments underscore the need to understand the underlying drivers of drug approvals at the level of the FDA [2, 5]. To address this knowledge gap, we analyzed a comprehensive collection of phase 3 oncologic RCTs.

## Methods

### Study design

**Fig. 1:**
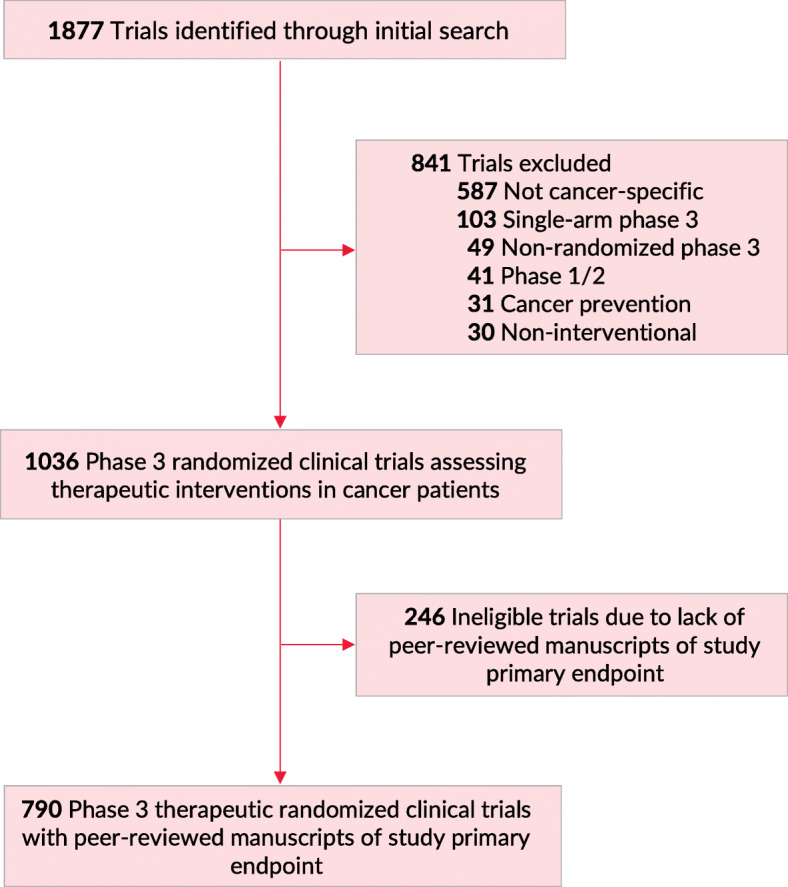
Flowchart of Trial Screening and Inclusion. Of 1877 trials identified on ClinicalTrials.gov (February 2020), 841 were excluded, for a final total of 1036 phase 3 randomized clinical trials assessing therapeutic interventions in patients with cancer. Of those, 790 trials had peer-reviewed manuscripts of primary study endpoint

We performed a database query through the ClinicalTrials.gov registry to search for oncologic phase 3 RCTs. Our query was performed on February 2020. The search was focused on cancer-specific, phase 3 RCTs that had reported results through the ClinicalTrials.gov registry. The following search parameters were used: terms: “cancer”; study type: “All Studies”; status: excluded “Not yet recruiting”; phase: phase 3; Study results: “With Results”. We screened all trials for therapeutic, cancer-specific, phase 3, randomized, multi-arm trials. Eligibility criteria were assessed through data from ClinicalTrials.gov, the trial’s protocol, and/or the primary publication of trial results (Fig. 1). Primary endpoints were classified into three categories: 1) overall survival, 2) quality of life metrics and/or patient reported outcomes, 3) other endpoints (including surrogate endpoints such as progression-free survival). Additional details on the search methodology are available in previous publications [6]. We identified whether a trial was used for subsequent FDA drug approval through screening of FDA approval announcements. FDA drug approvals were counted for new drugs, new indications, and new drug dosing. Both regular and accelerated approvals were included, irrespective of whether the trial was an FDA registration trial or not. No institutional review board approval was required as all data were publicly available without use of protected health information; no informed consent was required.

### Statistical analysis

We used Pearson’s *Chi-*squared testing for univariate analyses in testing the association between categorical trial-related factors and FDA approvals. Binary logistic regression was used for univariate analysis of continuous variables. Trial factors with two-sided *P*-value less than 0.05 on univariate analysis were subsequently included in multiple binary logistic regression for multivariable analysis. Statistical significance was set a priori at two-sided α = 0.05. All analyses were performed using IBM SPSS version 26.

## Results

In total, 790 trials were included in our study, with 225 trials (28.4%) generating data that were subsequently used for FDA approvals. Two-hundred and twenty of those trials (220/225, 97.8%) showed improvement in all primary endpoints analyzed. Of the 225 FDA approvals identified, 65 (28.9%) were based on trials assessing OS as a PEP, two (0.9%) were based on trials with a QoL PEP, and 158 approvals (70.2%) were based on trials with other PEP (*P =* 0.01) (Table 1). Of the 158 trials with other PEPs that led to FDA approvals, 96 trials (60.8%) assessed PFS as PEP. FDA approvals were more common among industry funded-trials (219/225, 97.3%; *P* < 0.001), and less common among trials sponsored by national cooperative groups (21/225, 9.3%; *P* < 0.001). Trials including advanced or metastatic solid tumors led to more FDA approvals (*P* < 0.001). Finally, increased *pre-hoc* power and meeting patients’ accrual target were associated with FDA approvals (*P* < 0.001). On multivariable analysis, industry support, cooperative-group sponsorship, line of therapy, intervention modality, meeting accrual target, and increased *pre-hoc* power were independently associated with subsequent FDA approval (Table 1).

**Table 1:** Trial variables associated with subsequent Food and Drug Administration drug approval

Trial Variables	FDA Approval (***N*** = 225)	No FDA Approval (***N*** = 565)	Univariate Analysis^f^	Multiple Binary Logistic Regression^f^	
	N (%)	N (%)	*P*	aOR [95%CI]	*P*
**Primary Endpoint**					
Overall Survival	65 (28.9)	162 (28.7)	0.01	–	
Quality of Life	2 (0.9)	41 (7.3)		0.4 [0.1–3.6]	0.41
Other Endpoints^a^	158 (70.2)	362 (64.1)		1.4 [0.9–2.1]	0.17
Progression-Free Survival	96 (42.7)	138 (24.4)			
Disease-Free Survival	8 (3.6)	39 (6.9)			
Event-Free Survival	2 (0.9)	10 (1.8)			
Complete Response	4 (1.8)	9 (1.6)			
Safety/Toxicity	1 (0.4)	28 (5.0)			
**Industry-Funding**^b^	219 (97.3)	387 (68.5)	< 0.001	5.9 [2.1–16.7]	0.01
**Cooperative-Group-Support**^b^	21 (9.3)	214 (37.9)	< 0.001	0.5 [0.2–0.9]	0.02
**Line of Therapy**					
Advanced/Metastatic First-Line	67 (29.8)	174 (30.8)	< 0.001	–	
Advanced/Metastatic Second-Line	67 (29.8)	104 (18.4)		1.0 [0.7–1.7]	0.85
Localized Solid	22 (9.8)	119 (21.1)		0.8 [0.4–1.6]	0.53
Hematological First-Line	35 (15.6)	67 (11.9)		2.5 [1.3–4.8]	0.01
Hematological Relapsed/Refractory	26 (11.6)	23 (4.1)		2.4 [1.2–4.8]	0.02
Mixed Stages	8 (3.6)	78 (13.8)		1.8 [0.5–7.2]	0.40
**Disease Site**					
Breast	35 (15.6)	112 (19.8)	0.07		
Gastrointestinal	24 (10.7)	74 (13.1)			
Genitourinary	30 (13.3)	65 (11.5)			
Head and Neck	7 (3.1)	21 (3.7)			
Hematologic	60 (26.7)	95 (16.8)			
Lungs	29 (12.9)	85 (15.0)			
**Modality**^c^					
Systemic Therapy^d^	212 (94.2)	409 (72.4)	< 0.001	–	
Radiation Therapy	0 (0)	23 (4.1)		0 [0-NA]	0.99
Surgery	0 (0)	8 (1.4)		0 [0-NA]	0.99
Supportive Care^e^	13 (5.8)	123 (21.8)		0.2 [0.1–0.7]	0.01
**Patient Accrual Met**	204 (91.9)	421 (76.5)	< 0.001	2.6 [1.4–4.7]	0.01
**Total Patients Enrolled** – Median [IQR]	572 [366–866] uOR: 1.0	449 [230–772]	0.97		
***Pre-Hoc*** **Power** – Median [IQR]	90 [80–90] uOR: 1.1	83 [80–90]	< 0.001	1.1 [1.05–1.14]	< 0.001

*Abbreviation*: *uOR* unadjusted Odds Ratio, *aOR* adjusted Odds Ratio, *CI* confidence interval, *IQR* interquartile range, *FDA* Food and Drug Administration

^a^ The most common primary endpoints used in phase 3 clinical trials other than overall survival and quality of life metrics were noted

^b^ Industry funding and cooperative group sponsorship were considered independent variables as certain trials were both industry-funded and performed through a multi-institutional cooperative group

^c^ Modality addressed the primary intervention as part of the randomization

^d^ Systemic therapy trials, including chemotherapy, targeted systemic agents, immunotherapy, and others, accounted for most trials by modality; they used systemic therapies to improve disease-related outcomes (eg, overall survival, disease-free survival)

^e^ Supportive care trials were those where the intervention aimed to reduce disease- or treatment-related toxic effects as the primary endpoint

^f^ Pearson’s *Chi-*squared and univariate binary logistic regression test were used in univariate analyses to assess the association between individual trial variables and subsequent FDA drug approval. Trial variables that had a two-sided *P*-value less than 0.05 were subsequently included in multiple binary logistic regression modelling

## Discussion

The current analysis shows that a majority of FDA approvals are using data generated from trials with surrogate PEPs (158/225 trials, 70.2%). It is worth noting that the FDA approvals analyzed in our study included both regular and accelerated approvals, and accelerated approvals are commonly based on surrogate endpoints, with a requirement for the sponsors to conduct post-market studies to confirm clinical benefit. Nevertheless, our results are consistent with prior data demonstrating increased utilization of surrogate endpoints as the basis for FDA drug approvals broadly across medical disciplines during the last few decades [7]. The use of surrogate endpoints has been suggested in clinical trials as it can facilitate FDA approvals and lead to rapid use of novel treatments [8, 9]. Furthermore, surrogate endpoints are often properly used in studies analyzing new drug indications or new drug dosing and schedule in drugs with previously established safety and efficacy profiles. To date, the associations between various surrogate oncologic endpoints and OS or QoL remains unclear [2]. A recent analysis of patients with breast, lung, pancreatic, and colorectal cancers showed an association between disease-free survival and QoL [10]. However, other studies analyzing the relationship between PFS and QoL showed a weak correlation between those two endpoints [3, 11]. Other studies demonstrated a weak correlation between PFS and OS, and a poor predictive value of PFS for OS, further questioning the validity of current surrogate endpoints [4, 12, 13]. In that context, the identification of valid surrogate endpoints in oncology is crucial to facilitate the execution of future trials [9]. We encourage utilization of non-surrogate PEPs when possible, in order to ensure that results directly impact patients’ quality and/or quantity of life. Furthermore, identifying valid surrogate endpoints is critical in the effort to more rapidly complete clinical trials and meaningfully advance the standard of care for patients with cancer [8, 9].

In the last few decades, the cancer research community has witnessed an increase in industry-funded trials, with approximately 80% of modern phase 3 cancer RCTs being funded by biopharmaceutical companies [6, 14]. This has certainly led to increased breakthroughs in cancer treatment [5]. Our data reveal remarkably higher rates of FDA drug approvals in trials receiving industry support. Moreover, cooperative-group-sponsored trials had lower rates of trial-associated FDA approvals. Of the 219 industry-funded trials that led to FDA approvals, 204 trials (93.2%) were funded solely by biopharmaceutical companies, and only 15 trials (6.8%) received co-sponsorship from national academic cooperative groups. Efforts to better understand the complexities in the interplay between the biopharmaceutical industry, academic cooperative groups, and regulatory agencies are critical.

When analyzing study design, our data show that increased *pre-hoc* statistical power was associated with FDA approvals. While a general consensus on *pre-hoc* power level is not clear and often dependent on the PEP analyzed, using higher power may entail a need for greater funding to ensure higher patient enrollment. Recent data have shown that industry-funding is associated with higher rates of clinical trials meeting their pre-specified patient accrual targets, while cooperative group sponsorship may be associated with accrual failure [15]. Further studies into the role of *pre-hoc* power, accrual success, and subsequent regulatory approval will be integral in determining optimal methods in improving trials’ design and execution among both industry-supported and cooperative-group-sponsored studies.

Our study is limited by the use of only one clinical trial registry, and one national regulatory body (FDA). Given that ClinicalTrials.gov represents the mandatory clinical trial registry for the United States, our results may not be generalizable elsewhere in the world. Furthermore, the registration of clinical trials on the ClinicalTrials.gov registry was not mandated until 2007, and so our study may underestimate the number of phase 3 oncologic trials leading to FDA approvals. Additionally, many phase 3 cancer RCTs are not designed to generate data leading to regulatory approval, and, therefore our analysis focused on comparisons between studies that did lead to FDA approval. Some approvals do not come from phase 3 trials, but rather may arise from phase 2 studies, or abstracts with preliminary results of phase 3 trials. We included both regular and accelerated FDA approvals, and no comparison between both types of approvals was performed. Lastly, many drugs require several months before acquiring FDA approval, and as such, our study may have missed some FDA approvals based on trials that published their results in 2020. Nevertheless, our study remains the largest to assess FDA approvals among phase 3 cancer trials.

## Conclusions

Our data show a high rate of FDA approvals from industry-funded trials. Furthermore, surrogate PEPs are common in phase 3 trials leading to subsequent FDA approvals. Additional studies are required to understand the complexity of FDA approvals and inform regulatory decisions to improve both the quality and quantity of life for patients with cancer.

## Data Availability

The data that support the findings of this study are available from J.A.J., R.K., E.B.L., and/or C.M.T., upon reasonable request.
